# Effects of clazosentan, cilostazol, and statins on aneurysmal subarachnoid hemorrhage: A protocol for systematic review and meta-analysis

**DOI:** 10.1097/MD.0000000000019902

**Published:** 2020-04-24

**Authors:** Junfang He, Li Zhang, Yao Yu, Xinyue Luo, Min Wei, Gen Chen, Yanfei Shen

**Affiliations:** a Department of Neurological Rehabilitation, Rehabilitation Center Hospital of Gansu Province; b The Third Ward of Cardiovascular Clinical Medical Center, Affiliated Hospital of Gansu University of Chinese Medicine; c Pathogens Biology Institute, School of Basic Medical Sciences of Lanzhou University; d The Second Clinical Medical College of Lanzhou University; e Affiliated Hospital of Gansu University of Chinese Medicine, Lanzhou; f Basic Medical School, Guilin Medical University, Guilin; g Gansu Provincial Hospital, Lanzhou, China.

**Keywords:** aneurysmal subarachnoid hemorrhage, cilostazol, clazosentan, network meta-analysis, statins

## Abstract

**Background:**

: Aneurysmal subarachnoid hemorrhage (aSAH) is a disease caused by the infiltration of blood into the subarachnoid space due to the rupture of an intracranial aneurysm. It is a serious cerebrovascular disease, with a mortality rate of about 40% worldwide, which seriously threatens human life and health. Many drugs are used to treat aSAH and its complications, and some have been tested in systematic reviews and have shown good effects. But which drug has the best effect remains unclear. This network meta-analysis (NMA) aims to assess the effectiveness and feasibility of clazosentan, cilostazol, and statins in patients with aSAH.

**Methods:**

: We will search for EMBASE.com, PubMed, the Cochrane Library, and Web of Science from inception to December 2019. Randomized controlled trials (RCTs) reporting efficacy and safety of clazosentan, cilostazol, and statins compared with the control, or compared with each other for the treatment of aSAH will be included. Two independent reviewers will assess the risk of bias of the included RCTs with the Cochrane “Risk of bias” tool. The pairwise meta-analysis will be performed with the random-effects model. The NMA will be performed in a Bayesian hierarchical framework using Markov Chain Monte Carlo method in WinBUGS 1.4.3. Egger test and funnel plot will be used to assess the publication bias. We will evaluate the quality of evidence for each outcome according to the GRADE approach.

**Results:**

: The results of this NMA will be submitted to a peer-reviewed journal for publication.

**Conclusion:**

: This study will summarize up-to-date evidence to compare the efficacy and safety of clazosentan, cilostazol, and statins on aSAH.

**PROSPERO registration number:** CRD42019147523.

## 1 Introduction

Aneurysmal subarachnoid hemorrhage (aSAH) is a disease caused by the infiltration of blood into the subarachnoid space due to the rupture of an intracranial aneurysm.^[1]^ It is a severe cerebrovascular disease, accounting for 5% of all strokes.^[2,3]^ Aneurysmal subarachnoid hemorrhage often causes many complications, such as cerebral ischemia, vasospasm, bacterial meningitis, cephaledema, hydrocephalus, and seizure.^[4–7]^ Despite advances in the diagnosis and treatment of intracranial aneurysms after decades of research, the incidence and mortality of aSAH are still high.^[8,9]^ In the United States, approximately 30,000 people suffer from aSAH each year, with a mortality rate of nearly 50%.^[10,11]^ Globally, the mortality of aSAH is about 40%,^[12]^ which seriously threatens human life and health.

Many drugs have been used to treat aSAH and its complications and have shown potential effects. Clazosentan, an endothelin receptor A antagonist, prevents angiographic vasospasm and delayed ischemic neurologic deficit (DIND), reduces all-cause mortality and vasospasm-related morbidity in patients with aSAH.^[13–15]^ Cilostazol can prevent thrombosis and dilate blood vessels and has a preventive effect on hydrocephalus secondary to aSAH.^[16,17]^ Statins (3-hydroxy-3-methylglutaryl coenzyme A reductase inhibitor) can increase the expression of nitric oxide synthase in the brain endothelium, improve endothelial function, increase cerebral blood flow, and prevent ischemia.^[18,19]^ Many meta-analyses have also evaluated the effects of these drugs on aSAH.^[20–22]^ But most of them are pairwise meta-analyses that compare the efficacy and safety of one drug with a placebo or another drug, and lack of studies comparing multiple interventions simultaneously. Therefore, it is unclear which drug has the best effect with less adverse effects.

Systematic reviews and meta-analyses are essential tools for generating reliable evidence of healthcare decisions.^[23–25]^ Network meta-analysis (NMA) is an extension of conventional meta-analyses of a given condition, which can be used to assess the relative effectiveness of multiple interventions and rank ordering of the interventions.^[26–28]^ Thus, we will perform a NMA to assess the effectiveness and feasibility of clazosentan, cilostazol, and statins in patients with aSAH.

## 2 Methods

This study protocol has been registered on PROSPERO (International Prospective Register of Systematic Reviews). The registration number is CRD42019147523. This NMA will be conducted and reported according to the Preferred Reporting Items for network meta-analyses (PRISMA-NMA) statement.^[29]^

### 2.1 Eligibility criteria

#### 2.1.1 Types of study

Randomized controlled trials (RCTs) reporting efficacy and safety of clazosentan, cilostazol, and statins compared with the control or compared with each other for the treatment of aSAH. RCTs should describe the number of patients and events of interest in each group. Cluster-randomized or quasi-randomized trials will be excluded.

#### 2.1.2 Participants

Adult patients (aged 18 years or older) diagnosed with aSAH, regardless of gender, ethnicity, and duration of illness.

#### 2.1.3 Interventions

Any dose of clazosentan, cilostazol, and statins. The intervention also can be a combination of these 3 or 2 drugs.

#### 2.1.4 Comparisons

The control will include clazosentan, cilostazol, statins, and placebo.

#### 2.1.5 Outcomes

The primary outcomes will include poor clinical outcome, DIND requiring rescue therapy, all-cause mortality, and vasospasm-related morbidity. The poor outcome is defined as the Glasgow outcome scale score of 1 to 3 or the modified Rankin Scale of 3 to 6 at the end of the follow-up.^[15,21]^ DIND is defined as a decrease of 2 or more points on the modified Glasgow Coma Scale or an increase of 2 or more points on the National Institutes of Health Stroke Scale lasting more than 2 hours.^[20]^ All-cause mortality is defined as death for any cause. Vasospasm-associated morbidity is defined as cerebral infarction or DIND associated with vasospasm and is not associated with rescue therapy.^[20]^ The secondary outcomes include severe angiographic cerebral vasospasm, symptomatic vasospasm, and new cerebral infarction.

### 2.2 Information sources

We will search EMBASE.com, PubMed, the Cochrane Library, and Web of Science from inception to December 2019. We will also retrieve the reference lists of published systematic reviews to identify additional trials.

### 2.3 Search strategy

A combination of subject terms and keywords will be used and we will make appropriate adjustments of vocabulary and grammar between different databases. The keywords include: “clazosentan,” “cilostazol,” “statins,” “aneurysm,” “subarachnoid hemorrhage,” “intracranial hemorrhage,” and “hemorrhagic stroke.” Search strategy of PubMed as follows:

#1 “Clazosentan” [Supplementary Concept] OR Clazosentan [Title/Abstract] OR VML-588 [Title/Abstract] OR VML 588 [Title/Abstract] OR VML588 [Title/Abstract] OR AXV-034343 [Title/Abstract] OR Ro 61–1790 [Title/Abstract] OR Ro-61–1790 [Title/Abstract] OR AXV 034 [Title/Abstract] OR AXV-034 [Title/Abstract] OR AXV 343434 [Title/Abstract] OR AXV343434 [Title/Abstract] OR AXV-343434 [Title/Abstract]

#2 “Cilostazol” [Mesh] OR 6-(4-(1-Cyclohexyl-1H-tetrazol-5-yl)butoxy)-3,4-dihydro-2(1H)-quinolinone [Title/Abstract] OR OPC 13013 [Title/Abstract] OR OPC-13013 [Title/Abstract] OR Pletal [Title/Abstract]

#3 “Hydroxymethylglutaryl-CoA Reductase Inhibitors” [Mesh] OR Hydroxymethylglutaryl-CoA Reductase Inhibitors [Title/Abstract] OR Hydroxymethylglutaryl CoA Reductase Inhibitors [Title/Abstract] OR HMG-CoA Reductase Inhibitors [Title/Abstract] OR HMG CoA Reductase Inhibitors [Title/Abstract] OR Hydroxymethylglutaryl-CoA Inhibitors [Title/Abstract] OR Hydroxymethylglutaryl CoA Inhibitors [Title/Abstract] OR Hydroxymethylglutaryl-Coenzyme A Inhibitors [Title/Abstract] OR Hydroxymethylglutaryl Coenzyme A Inhibitors [Title/Abstract] OR Statins [Title/Abstract] OR Statin [Title/Abstract] OR Lovastatin [Title/Abstract] OR Pravastatin [Title/Abstract] OR Simvastatin [Title/Abstract] OR Fluvastatin [Title/Abstract] OR Atorvastatin[Title/Abstract] OR Rosuvastatin [Title/Abstract] OR Pitavastatin [Title/Abstract]

#4 #1 OR #2 OR #3

# 5 “Subarachnoid Hemorrhage” [Mesh] Subarachnoid Hemorrhage [Title/Abstract] OR Subarachnoid Hemorrhages [Title/Abstract] OR SAH[Title/Abstract] OR SAHs [Title/Abstract] OR aSAH [Title/Abstract] OR aSAHs [Title/Abstract] OR Aneurysmal Subarachnoid Hemorrhage [Title/Abstract] OR Aneurysmal Subarachnoid Hemorrhages [Title/Abstract] OR Intracranial Hemorrhage [Title/Abstract] OR Intracranial Hemorrhages [Title/Abstract] OR Hemorrhagic Stroke [Title/Abstract]

#6 #4 AND #5

### 2.4 Selection of studies

Reference management software (Endnote X8) will be used to manage the identified records. We will first remove potential duplicate records. Then, 2 independent reviewers will read the title or abstract, or both of each record, to determine which studies should be assessed further. We will investigate all potentially relevant records as full text and classify studies as included studies, excluded studies, studies awaiting classification or ongoing studies in accordance with the inclusion and exclusion criteria. The reasons for the excluded studies will be recorded. We will resolve any discrepancies through consensus or recourse to a third review author. If there are studies with overlapping data, we will choose a study with larger sample size. For studies that the information is incomplete or not reported, we will attempt to contact authors for more information.

### 2.5 Data extraction

Data extraction forms with detailed written instructions will be used to collect pertinent information and data. The detailed information includes author, years of publication, country, language, setting, age, gender, ethnicity, number of participants, participants lost to follow-up, treatment modalities, dose of treatment, duration of treatment, and duration of follow-up. One author will extract data from the included studies and a second author will check the data. Disagreements will be resolved by consensus, or by involving a third reviewer.

### 2.6 Assessment of risk of bias

Two independent reviewers will assess the risk of bias of the included RCTs with the Cochrane “Risk of bias” tool.^[30]^ We will resolve any disagreement by discussion or by involving a third review author. The tool consists of random sequence generation, allocation concealment, blinding of all participants and personnel, blinding of outcome assessment, incomplete outcome data, selective reporting, and other sources of bias. We will judge the risk of bias domains as low, high, or unclear risk.

### 2.7 Geometry of the evidence network

We will create network diagrams to describe and present the geometry of the intervention network of comparisons across trials using STATA (13.0; Stata Corporation, College Station, TX). For studies that are not linked by interventions, we excluded them from the NMA and only describe the findings. In the network diagram, nodes represent different interventions, and edges represent head-to-head comparisons between interventions. The size of the nodes indicates the sample size of the intervention, and the thickness of the edges indicates the number of trials included.

### 2.8 Statistical analysis

#### 2.8.1 Measures of treatment effect

For dichotomous variables, we will calculate the pooled risk ratios with the 95% confidence intervals (95% CIs). For continuous variables, we will calculate the mean differences or standardized mean differences (if different studies use different measures to assess the same outcome) with 95% CIs to express the overall effect.

#### 2.8.2 Data synthesis

We will first perform pairwise meta-analyses to estimate the effect of different interventions with the random-effects model based on the DerSimonian and Laird method adjusted by the Knapp–Hartung method.^[31]^ The NMA will be performed in a Bayesian hierarchical framework using Markov Chain Monte Carlo method in WinBUGS 1.4.3 (MRC Biostatistics Unit, Cambridge University, UK).^[32]^ The model fit and parsimony will be evaluated using the deviance information criterion (DIC). The convergence will be assessed using the Brooks-Gelman-Rubin (BGR) plots method.^[33]^ We will use the surface under the cumulative ranking curve (SUCRA) to rank the treatments according to each outcome accounting for the uncertainty in the treatment effects. The absolute rank of the treatment per outcome is presented using ’Rankograms’ that visually show the distribution of ranking probabilities.^[34]^ The node splitting method will be adopted to examine the inconsistency between direct and indirect comparisons if a loop connecting 3 or more arms exist.^[35]^ We will create all the result figures using STATA (13.0; Stata Corporation, TX) software.

#### 2.8.3 Assessment of heterogeneity

Statistical heterogeneity within each pairwise comparison will be assessed using the I^2^ statistic, and I^2^ values of 25%, 50%, and 75% represent low, moderate, and severe statistical heterogeneity, respectively.^[31]^ We will explore sources of heterogeneity by subgroup analyses and meta-regression analyses. If clinical heterogeneity is present, the pairwise comparison will not be included in the NMA.

#### 2.8.4 Subgroup analysis

If the necessary data are available, subgroup analyses will be done based on gender, country, age, and dose of interventions.

#### 2.8.5 Sensitivity analysis

Sensitivity analyses by sequentially excluding one study at a time or by removing low-quality studies will be carried out to check whether the results are robust.

#### 2.8.6 Assessment of publication bias

Egger test and funnel plot will be performed to assess the publication bias when applicable.

### 2.9 Quality of evidence

We will evaluate the overall quality of the evidence for primary and secondary outcomes according to the Grading of Recommendations Assessment, Development, and Evaluation (GRADE) approach. The GRADE tool consists of 5 criteria: risk of bias, publication bias, imprecision, inconsistency, and indirectness.^[36]^ For each outcome, 2 review authors will independently rate the quality of evidence for each comparison as high, moderate, low, or very low using GRADEpro GDT. Any discrepancies will be resolved by consensus or by arbitration with a third review author. All results will be presented in a 'Summary of findings’ table.

## 3 Ethics and dissemination

Ethics approval and patient consent are not required as this study is a NMA based on published trials. This study will compare the efficacy and safety of clazosentan, cilostazol, and statins on aSAH. The results of this NMA will be submitted to a peer-reviewed journal for publication. We hope the findings of this study will help clinicians and patients to select an optimal drug for patients with aSAH.

## Author contributions

JFH, LZ, and MW planned and designed the research. JFH, LZ, YY, and XYL tested the feasibility of the study. MW, GC, and YFS provided methodological advice, polished and revised the manuscript. JFH, LZ, and MW wrote the manuscript. All authors approved the final version of the manuscript.

**Conceptualization:** Junfang He, Li Zhang, Xinyue Luo.

**Funding acquisition:** Yanfei Shen.

**Investigation:** Li Zhang, Yao Yu.

**Methodology:** Junfang He, Min Wei.

**Project administration:** Min Wei.

**Resources:** Junfang He, Li Zhang, Yao Yu, Xinyue Luo.

**Supervision:** Min Wei.

**Validation:** Min Wei, Gen Chen, Yanfei Shen.

**Visualization:** Junfang He, Yao Yu.

**Writing – original draft:** Junfang He, Li Zhang, Min Wei.

**Writing – review & editing:** Junfang He, Li Zhang, Min Wei.

## References

[1] Chen TF Chen KW Chien Y Dental pulp stem cell-derived factors alleviate subarachnoid hemorrhage-induced neuroinflammation and ischemic neurological deficits. Int J Mol Sci 2019;20:3747. 10.3390/ijms20153747 PMC6695587 31370244

[2] Liu ZW Zhao J Pang HG Vascular endothelial growth factor A promotes platelet adhesion to collagen IV and causes early brain injury after subarachnoid hemorrhage. Neural Regen Res 2019;14:1726–33. 31169190 10.4103/1673-5374.257530 PMC6585561

[3] Macdonald RL Delayed neurological deterioration after subarachnoid haemorrhage. Nat Rev Neurol 2014;10:44–58. 24323051 10.1038/nrneurol.2013.246

[4] Diringer MN Bleck TP Claude Hemphill J 3rd Critical care management of patients following aneurysmal subarachnoid hemorrhage: recommendations from the neurocritical care society's multidisciplinary consensus conference. Neurocritical care 2011;15:211–40. 21773873 10.1007/s12028-011-9605-9

[5] Keyrouz SG Diringer MN Clinical review: prevention and therapy of vasospasm in subarachnoid hemorrhage. Crit Care 2007;11:220. 17705883 10.1186/cc5958 PMC2206512

[6] Schlenk F Frieler K Nagel A Cerebral microdialysis for detection of bacterial meningitis in aneurysmal subarachnoid hemorrhage patients: a cohort study. Crit Care 2009;13:R2. 19154580 10.1186/cc7689 PMC2688112

[7] Ling GQ Li XF Lei XH c-Jun N-terminal kinase inhibition attenuates early brain injury induced neuronal apoptosis via decreasing p53 phosphorylation and mitochondrial apoptotic pathway activation in subarachnoid hemorrhage rats. Mol Med Rep 2019;19:327–37. 30431087 10.3892/mmr.2018.9640 PMC6297759

[8] Can A Lai PMR Castro VM Decreased total iron binding capacity may correlate with ruptured intracranial aneurysms. Sci Rep 2019;9:6054. 30988354 10.1038/s41598-019-42622-y PMC6465340

[9] Vlak MH Algra A Brandenburg R Prevalence of unruptured intracranial aneurysms, with emphasis on sex, age, comorbidity, country, and time period: a systematic review and meta-analysis. Lancet Neurol 2011;10:626–36. 21641282 10.1016/S1474-4422(11)70109-0

[10] Jones S Schwartzbauer G Jia X Brain monitoring in critically neurologically impaired patients. Int J Mol Sci 2016;18:43. 10.3390/ijms18010043 PMC5297678 28035993

[11] Zacharia BE Hickman ZL Grobelny BT Epidemiology of aneurysmal subarachnoid hemorrhage. Neurosurg Clin N Am 2010;21:221–33. 20380965 10.1016/j.nec.2009.10.002

[12] Lindbohm JV Rautalin I Jousilahti P Physical activity associates with subarachnoid hemorrhage risk- a population-based long-term cohort study. Sci Rep 2019;9:9219. 31239477 10.1038/s41598-019-45614-0 PMC6592878

[13] Thampatty BP Sherwood PR Gallek MJ Role of endothelin-1 in human aneurysmal subarachnoid hemorrhage: associations with vasospasm and delayed cerebral ischemia. Neurocrit Care 2011;15:19–27. 21286855 10.1007/s12028-011-9508-9 PMC3134137

[14] Shen J Pan JW Fan ZX Dissociation of vasospasm-related morbidity and outcomes in patients with aneurysmal subarachnoid hemorrhage treated with clazosentan: a meta-analysis of randomized controlled trials. J Neurosurg 2013;119:180–9. 23641823 10.3171/2013.3.JNS121436

[15] Fujimura M Joo JY Kim JS Preventive effect of clazosentan against cerebral vasospasm after clipping surgery for aneurysmal subarachnoid hemorrhage in Japanese and Korean patients. Cerebrovasc Dis 2017;44:59–67. 28463833 10.1159/000475824 PMC5475234

[16] Junichi K Yongge L Bing S Cilostazol as a unique antithrombotic agent. Curr Pharm Des 2003;9:2289–302. 14529391 10.2174/1381612033453910

[17] Nakatsuka Y Kawakita F Yasuda R Preventive effects of cilostazol against the development of shunt-dependent hydrocephalus after subarachnoid hemorrhage. J Neurosurg 2016;127:319–26. 27494819 10.3171/2016.5.JNS152907

[18] Sugawara T Ayer R Zhang JH Role of statins in cerebral vasospasm. Acta Neurochir Suppl 2008;104:287–90. 18457003 10.1007/978-3-211-75718-5_59 PMC2743544

[19] McGirt MJ Lynch JR Parra A Simvastatin increases endothelial nitric oxide synthase and ameliorates cerebral vasospasm resulting from subarachnoid hemorrhage. Stroke 2002;33:2950–6. 12468796 10.1161/01.str.0000038986.68044.39

[20] Cho SS Kim SE Kim HC Clazosentan for aneurysmal subarachnoid hemorrhage: an updated meta-analysis with trial sequential analysis. World Neurosurg 2019;123:418–24. e3. 30508597 10.1016/j.wneu.2018.10.213

[21] Shan T Zhang T Qian W Effectiveness and feasibility of cilostazol in patients with aneurysmal subarachnoid hemorrhage: a systematic review and meta-analysis. J Neurol 2019;[Epub ahead of print] doi: 10.1007/s00415-019-09198-z. 10.1007/s00415-019-09198-z 30739182

[22] Su SH Xu W Hai J Effects of statins-use for patients with aneurysmal subarachnoid hemorrhage: a meta-analysis of randomized controlled trials. Sci Rep 2014;4:4573. 24763190 10.1038/srep04573 PMC5381185

[23] Gao Y Li J Ma X The value of four imaging modalities in diagnosing lymph node involvement in rectal cancer: an overview and adjusted indirect comparison. Clin Exp Med 2019;19:225–34. 30900099 10.1007/s10238-019-00552-z

[24] Tian JH Zhang J Ge L The methodological and reporting quality of systematic reviews from China and the USA are similar. J Clin Epidemiol 2017;85:50–8. 28063911 10.1016/j.jclinepi.2016.12.004

[25] Gao Y Cai Y Yang K Methodological and reporting quality in non-Cochrane systematic review updates could be improved: a comparative study. J Clin Epidemiol 2020;119:36–46. 31759063 10.1016/j.jclinepi.2019.11.012

[26] Mills EJ Thorlund K Ioannidis JP Demystifying trial networks and network meta-analyses. BMJ 2013;346:f2914. 23674332 10.1136/bmj.f2914

[27] Gao Y Ge L Ma X Improvement needed in the network geometry and inconsistency of Cochrane network meta-analyses: a cross-sectional survey. J Clin Epidemiol 2019;113:214–27. 31150834 10.1016/j.jclinepi.2019.05.022

[28] Li L Tian J Tian H Network meta-analyses could be improved by searching more sources and by involving a librarian. J Clin Epidemiol 2014;67:1001–7. 24841794 10.1016/j.jclinepi.2014.04.003

[29] Hutton B Salanti G Caldwell DM The PRISMA extension statement for reporting of systematic reviews incorporating network meta-analyses of health care interventions: checklist and explanations. Ann Intern Med 2015;11:777–84. 10.7326/M14-2385 26030634

[30] Higgins JP Altman DG Gotzsche PC The Cochrane Collaboration's tool for assessing risk of bias in randomised trials. BMJ 2011;343:d5928. 22008217 10.1136/bmj.d5928 PMC3196245

[31] Collet C Asano T Miyazaki Y Late thrombotic events after bioresorbable scaffold implantation: a systematic review and meta-analysis of randomized clinical trials. Eur Heart J 2017;38:2559–66. 28430908 10.1093/eurheartj/ehx155

[32] Lunn DJ Thomas A Best N WinBUGS-A Bayesian modeling framework: concepts, structure, and extensibility. Stat Comput 2000;10:325–37.

[33] Zhou X Zhang Y Furukawa TA Different types and acceptability of psychotherapies for acute anxiety disorders in children and adolescents: a network meta-analysis. JAMA Psychiatry 2019;76:41–50. 30383099 10.1001/jamapsychiatry.2018.3070 PMC6583467

[34] Salanti G Ades AE Ioannidis JP Graphical methods and numerical summaries for presenting results from multiple-treatment meta-analysis: an overview and tutorial. J Clin Epidemiol 2011;64:163–71. 20688472 10.1016/j.jclinepi.2010.03.016

[35] Dias S Welton NJ Caldwell DM Checking consistency in mixed treatment comparison meta-analysis. Stat Med 2010;29:932–44. 20213715 10.1002/sim.3767

[36] Puhan MA Schünemann HJ Murad MH A GRADE Working Group approach for rating the quality of treatment effect estimates from network meta-analysis. BMJ 2014;349:g5630. 25252733 10.1136/bmj.g5630

